# A review of gamified approaches to encouraging eco-driving

**DOI:** 10.3389/fpsyg.2022.970851

**Published:** 2022-09-02

**Authors:** Richard Stephens

**Affiliations:** School of Psychology, Keele University, Keele, United Kingdom

**Keywords:** review, gamification, eco-driving, flow, enjoyment

## Abstract

Eco-driving is a style of driving that minimizes energy consumption, while gamification refers to the use of game techniques to motivate user engagement in non-game contexts. This paper comprises a literature review assessing applying gamification to encourage eco-driving. The Web of Science Core Collection and EBSCO Host platforms were searched in February 2022. Qualifying sources included peer review journal articles, conference proceedings papers, academic book chapters and dissertation reports. The final sample comprised 39 unique publications, of which 34 described gamification adjunct systems used during driving. Most were designed as smartphone apps, but some ran on bespoke in-car feedback displays. Alternatively, using game-based learning, 5 studies described videogames designed to encourage eco-driving. Popular gamification elements were: an eco-driving score; self-comparisons or comparisons with others via leader boards; rewards; challenges, missions or levels; and emotive feedback (e.g., emojis). One system aimed to discourage driving at busy times. While 13 studies assessed the efficacy of the various systems, these were generally of poor quality. This developing literature contains many good ideas for applying gamification to promote eco-driving. However, evidence for efficacy is largely absent and researchers are encouraged to continue to evaluate a wide range of gamification approaches to promote eco-driving.

## Introduction

Eco-driving is a style of vehicle driving that reduces energy consumption, maximizing mileage per unit of energy consumed (Stillwater and Kurani, 2013). Eco-driving requires adherence to speed limits, accelerating and braking smoothly, avoiding over revving the engine, use of engine braking and maintaining a constant speed (Magaña and Muñoz-Organero, 2015). Eco-driving may also include vehicle maintenance, trip planning, switching to other transport where appropriate and vehicle choice (Stillwater and Kurani, 2013). Eco-driving may save up to 25% of fuel (Kamal et al., 2011). There is overlap between eco-driving and safe driving since safe driving entails observing speed limits and avoiding harsh acceleration and braking (Vaezipour et al., 2019).

Gamification refers to use of game techniques in non-game contexts to motivate user engagement (Diewald et al., 2013). Gamification provides intrinsic motivation for a behavior by virtue of rewards related to gameplay, such as attaining a target score, as opposed to extrinsic rewards like cash. McGonigal (2011) specifies four elements of intrinsic motivation: satisfying work with clear goals and tasks; hopes and/or experiences of success; social connection; and meaning. There are some overlaps between these elements and psychological flow theory (Csikszentmihalyi and LeFevre, 1989; Šimleša et al., 2018). The flow state is inherently enjoyable and is experienced when there is a good match between the challenges presented by a situation and the skills a person possesses to meet such challenges. Flow will not, however, be experienced for challenges that are too easy or difficult, and neither are these likely to be perceived as enjoyable. In the context of eco-driving, gamification may generate flow and consequent intrinsic motivation and enjoyment by increasing the level of challenge of the otherwise mundane task of driving.

Diewald et al. (2013) reviewed gamification in relation to driving. However, the eco-driving section was short, referenced few peer reviewed studies and an update has become timely. This paper comprises a systematized literature review (Grant and Booth, 2009) assessing using gamification to influence eco-driving. The aims of the review were (i) to summarize elements of gamification applied to eco-driving in the literature; (ii) to assess evidence of efficacy of gamified eco-driving solutions across shorter and longer time scales; and (iii) to assess user experience evaluations of the various gamified approaches.

## Methods

Searches were carried out in February 2022 in the Web of Science Core Collection and the EBSCO Host databases: Computer Science/Engineering Databases, Psychology and Sociology; Library, Information Science & Technology Abstracts; Academic Search Complete; eBook Collection (EBSCOhost); MEDLINE, APA PsycInfo, AgeLine, CINAHL Plus with Full Text.

The search term including Boolean operators was: “gamification” or “gamified” or “gameful” or “serious games” or “game-based learning” or “game” or “competition” or “competitive” or “leader board” or “leaderboard” and “ecodriving” or “eco-driving” or “eco driving” or “energy-efficient driving” or “energy efficient driving” or “low impact driving” or “green driving” or “safe driving.”

Study period protocol: Source publication year was open. Included publications spanned the period 2003–2021.

### Inclusion

Due to the relatively small size of this developing literature a wide variety of publication types was specified. Qualifying publication types were: peer review journal articles, conference proceedings papers, chapters in academic books and dissertation reports. Studies were included if they presented any gamification concept applied to eco-driving even if these terms were not mentioned. For example, a study mentioning “competition” but not “gamification” qualified for inclusion; a study mentioning “safe driving” qualified for inclusion where relevant behaviors, such as avoiding speeding, were mentioned. “Game-based learning” approaches also qualified for inclusion.

### Exclusion

Sources were excluded if no specific intervention or system was specified, if the content duplicated content already included from another source, or where the focus was on software design rather than application.

### Data collection process

**FIGURE 1:**
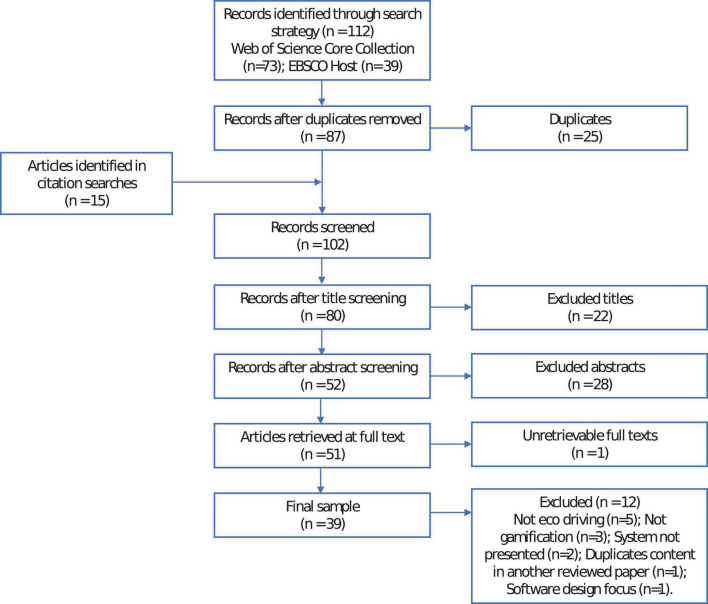
PRISMA diagram.

This is summarized in the PRISMA diagram (see Figure 1). Data were extracted by the author.

Research quality was assessed study-by-study in the results narrative.

## Results

### Elements of gamification applied to eco-driving in the literature

Of the final sample of 39 papers, 34 described a gamification solution for eco-driving in the form of an adjunct system designed for use while driving (see Table 1). These 34 papers describe 25 different adjunct systems. Most were designed as standalone smartphone apps (*n* = 12), some ran on bespoke dashboard feedback displays (*n* = 8), and others combined these formats (*n* = 3). Some used email and websites to convey feedback and gamified outcomes (*n* = 2).

**TABLE 1:** Details of adjunct systems (*n* = 25) and game-based learning systems (*n* = 5).

Name of system	References	Theoretical basis	Mode	During driving?	Common driving elements	Distinctive driving element	Eco driving score	Leader board	Personal Best	Reward	Missions/ Levels/Quests/ Challenges	Other
**Adjunct systems**												
GAFU/Eco driving assistant	Magaña and Muñoz-Organero, 2015, 2014, 2013	-	Smartphone	Y	a,b,c,d,f	-	Abstract	Y	-	Non-fiscal	-	-
Coastmaster; Brakemaster	Steinberger et al., 2017a,b	Opportunity Cost Model	Smartphone	Y	a,c,e	-	-	-	-	-	Y	-
-	Vaezipour et al., 2019; Vaezipour et al., 2016	User Centered Design	Bespoke screen	Y	a,b,c	-	Abstract	Y	-	Fiscal	Y	Happy/sad face; green/red light
GamECAR	Nousias et al., 2019; Tselios et al., 2019; Gardelis et al., 2018	Octalysis Actionable Gamification Framework	Smartphone	Y	a,b,c,d,e	-	Abstract	Y	Y	Non-fiscal	Y	-
Green Drive	Belotti et al., 2019; Dange et al., 2017; Paranthaman et al., 2016	Serious Games Community Building	Smartphone	Y	a,b,c,d,f	-	Energy use	Y	-	Fiscal	Y	Snakes and ladders
-	Stillwater and Kurani, 2013; Stillwater, 2011	-	Bespoke screen	Y	f,g	Cost per km	Energy use	Y	Y	-	Y	-
-	Gunther et al., 2020	-	Smartphone	N	f	-	Energy use	Y	-	Fiscal	-	-
-	Soriguera and Miralles, 2016	-	Smartphone	N	b,c	-	Abstract	Y	-	-	-	-
Project Drive	Bahadoor and Hosein, 2016	-	Smartphone	Y	a,b	-	Abstract	-	-	Non-fiscal	-	User stories posted on a social feed
The Eco Service	Rapp, 2016	-	Smartphone	N	a,b,c,f	Avoid idling time	Abstract	Y	Y	-	-	-
Driving Miss Daisy	Shi et al., 2012	-	Smartphone	Y	a,b,c		Abstract	Y	Y	-	Y	-
I-GEAR	McCall and Koenig, 2012	Persuasive gaming	Smartphone	N	-	Discourage driving altogether	-	-	-	Fiscal	-	
TEGA	Klemke et al., 2014	-	Smartphone	Y	a,d,f,g	-	Abstract	Y	-	Non-fiscal	Y	-
GreenDriver	Degirmenci, 2018	Gamification objects and mechanics	Smartphone	Y	b,c,f	-	Energy use	Y	-	Non-fiscal	Y	-
EcoChallenge	Ecker et al., 2011	Persuasive games	Dashboard display	Y	a,b,c,d,e,f	-	Abstract	Y	Y	-	Y	-
Metaphors	Beloufa et al., 2019	Cognitive theory of multimedia learning	Dashboard display	Y	a,b,c,d,g	Avoid idling	CO_2_	-	Y	-	-	-
-	Tractinsky et al., 2011	-	Dashboard display	Y	f	-	Energy use	Y	-	-	-	-
-	Rodríguez et al., 2014	Fogg Behavioral model	Smartphone/HUD/ mirror display	Y	a,c	-	Abstract	Y	-	Non-fiscal	-	-
ecoDriver	Brouwer et al., 2015	Value Orientation Theory	Bespoke screen	Y	a	-	Abstract	Y	-	-	Y	-
Social Driving App	Reiner and Reder, 2014	-	Bespoke screen	Y	a,d,f	-	Abstract	Y	-	-	-	-
-	Sundström et al., 2012	-	Bespoke screen	Y	b,c	-	-	-	-	-	-	Emotional car character
-	Loumidi et al., 2011	-	Dashboard display/Smartphone	Y	a,b,c,f,g	Cost per km	Abstract	Y	Y	-	-	Tree graphics
Autopet/Message-massage	Krome et al., 2014	-	Smartphone/massage pad	Y	a,b,c	-	-	-	-	-	-	Creature character depends on driving style/Seat massager relieves stress
-	Ando et al., 2010	-	Email and website	N	a,b,c,g	Avoid idling time, use of A/C, check tire pressure, minimise cargo	Abstract	Y	-	-	-	-
-	McConky et al., 2018	-	Website	N	a,b,c	-	CO_2_	Y	-	Fiscal	-	-
**Game-based learning systems**												
-	Rodrigues et al., 2015	Game Based Learning	Video game	N	a		Abstract	-	-	-	-	-
Streetwise	Bingham and Shope, 2003	-	Video game	N	a,b		-	-	-	-	Y	-
Meeco	Vara et al., 2011	-	Smartphone	N	-	Discourage driving altogether	-	-	-	Fiscal		Game people out of their cars altogether
iCO2	Hollerit et al., 2021; Prendinger et al., 2014	Games With a Purpose	Smartphone	N	a,b,c,f		Energy use	Y	-	Non-fiscal	Y	Time sensitive decisions

Common driving elements: speed (a); acceleration (b); braking (c); RPM/gear changes (d); coasting (e); energy/fuel use (f); CO_2_ emissions (g).

**FIGURE 2:**
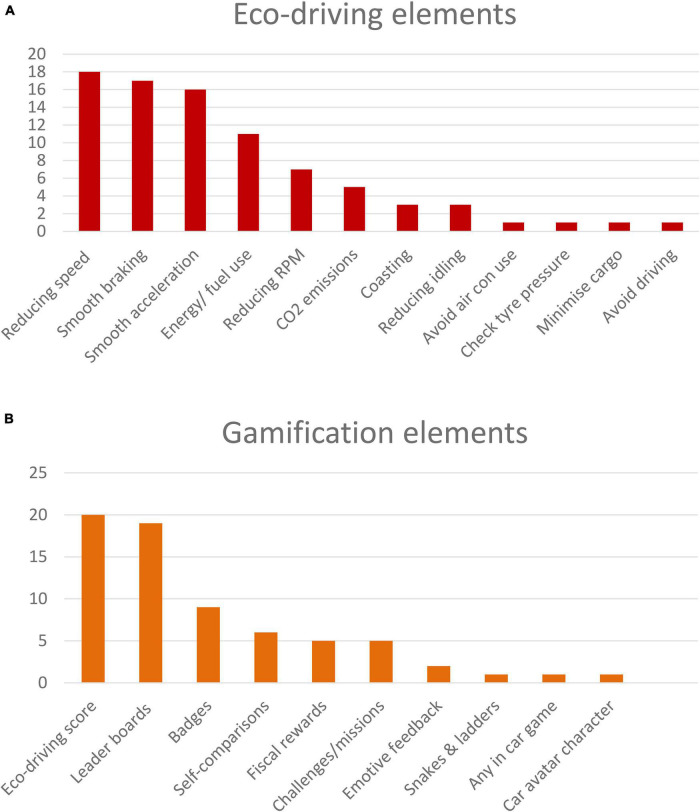
Eco-driving elements **(A)** and gamification elements **(B)** deployed across the 34 adjunct studies.

Eco-driving behaviors encouraged were: reducing speed (*n* = 18); smooth braking (*n* = 17); smooth acceleration (*n* = 16); avoiding excessive RPM (*n* = 7); coasting (*n* = 3); avoiding excessive idling (running the engine while stationary; *n* = 3); avoiding use of air conditioning (*n* = 1); checking tire pressure (*n* = 1); minimizing cargo (*n* = 1); avoiding driving (*n* = 1). Some adjunct systems logged energy or fuel use (*n* = 11) or CO2 emissions (*n* = 5). These are illustrated in Figure 2A.

Gamification elements were: an abstract eco-driving score (*n* = 20), a concrete eco-driving score such as miles per gallon, miles per kWh, or CO2 emissions reduction (*n* = 4); comparisons with others via leader boards (*n* = 19); self-comparisons (*n* = 6); non-fiscal rewards such as badges (*n* = 9); fiscal rewards such as restaurant vouchers (*n* = 5); challenges, missions, quests or levels (*n* = 5); emotive feedback such as happy/sad face emojis (*n* = 1), graphic trees that flourish or whither dependent on eco-driving (*n* = 1); connecting eco-driving to progress on the traditional board game snakes and ladders (*n* = 1); a car avatar that appears happy or sad dependent on eco-driving the real car (*n* = 1); a massage system to relieve stress in traffic jams (*n* = 1). Gamification elements such as feedback were usually presented during driving (*n* = 14), after driving (*n* = 8), both during and after driving (*n* = 2), or the timing was not clearly specified (*n* = 1). These are illustrated in Figure 2B.

Alternatively, five studies described four game-based learning systems comprising videogames designed to encourage eco-driving (see Table 1). Three of these videogames were driving simulations with elements of eco-driving promoted within. Two ran on desktop PCs, while the third was accessed on a smartphone. The eco-driving behaviors encouraged were: reducing speed (*n* = 3), smooth acceleration (*n* = 2), smooth braking (*n* = 1) and feeding back energy use (*n* = 1). The fourth game, delivered via smartphone, comprised a prompt system designed to nudge drivers off the road at busy times, for example by encouraging a driver to delay an intended car journey until after rush hour. Gamification elements were: an abstract eco-driving score (*n* = 2); challenges and levels (*n* = 2); a leader board (*n* = 1); non-fiscal rewards (*n* = 1), and time sensitive decisions and randomness interfering with progress (*n* = 1). Gamification elements were usually presented during simulated driving (*n* = 3).

### Evidence of efficacy of gamified eco-driving solutions that have been trialed, across shorter and longer time scales

Efficacy evaluation studies have been carried out for 12 of the 25 adjunct systems and one of the four game-based systems (see Table 2). Magaña and Muñoz-Organero (2015) evaluated a smartphone-based adjunct system across 36 drivers over 2,160 road trips in Spain. Only this latest iteration of the data set is reviewed here, although some of the same data appear to be presented in Magaña and Muñoz-Organero (2013) and Magaña and Muñoz-Organero (2014). Within each city, six drivers used the app on a setting which provided instant feedback of eco-driving scores, in-game achievements and social comparisons, while six further drivers used the app set to display only speed limit alerts, as a control group. Presented analyses in the form of *t*-tests comparing the means of the experimental and control groups in each city, appear to have been performed incorrectly, utilizing 60 data points per driver, which violates the assumption of data independence underlying parametric statistical analysis. A re-analysis of the performance means in Tables 7–9 (p. 67) showed that following 60 trips when the experimental group had the app running, average fuel consumption (l/100 km) was lower by 0.59 compared with controls, *t*(34) = 3.078, *p* = 0.004, *d* = 1.026. Based on this re-analysis, this paper provides good evidence for the efficacy of a gamified app for encouraging eco-driving.

**TABLE 2:** Evaluation studies.

References	Name of system	Setting	Sample N	Sample info	Design	Control condition	Length of evaluation	Effects
**Evaluations of efficacy**								
Gunther et al., 2020	NA	Road	108	28f 80 m	Within	Yes	22 months (6 months with gamification)	Reduced average energy consumption (2.99 kwh/100 km)
Ando et al., 2010	NA	Road	50	No details	Within	No	18 weeks	No analysis carried out
Stillwater, 2011	NA	Road	42	Varying age and sex	Within	Yes	4 weeks (2 weeks with gamification)	No overall effect
Magaña and Muñoz-Organero, 2015	GAFU/Eco driving assistant	Road	36	No details	Between	Yes	12 weeks	Average fuel consumption lower by 0.59 l/100 km
Ecker et al., 2011	Eco-Challenge	Road	36	Mostly male, aged 21–59 years	Within	Yes	1 h	Higher eco score, lower braking force, greater coasting mode, more speed variability, higher acceleration.
Rapp, 2016	The eco service	Road	16	Mixed gender; age 21–65	Between	Yes	1 h	No effect for fuel consumption and no analyses for other dependent variables
Soriguera and Miralles, 2016	NA	Road	7	1f 6 m, aged 26–57, drivers and motorcyclists	Within	No	Average 5.5 h driving per participant	No significant effects shown
Vaezipour et al., 2019	NA	Sim	40	20f, aged 18–65	Within	Yes	1 h	Reduced fuel consumption, less variability in accelerator pedal position and lower mean speed in 60 km/h speed zones.
Steinberger et al., 2017a	Coast-master	Sim	32	Male, aged 18–25	Within	Yes	16 min	Reduced overall speed, reduced speeding, greater anticipation
Steinberger et al., 2017b	Coast-master	Sim	24	Male, aged 18–25	Within	Yes	20 min	Lower average speed
McConky et al., 2018	NA	Sim	29	Mixed gender, young adult	Between	Yes	15 min	No effects of gamification compared to training
Brouwer et al., 2015	NA	Sim	26	2f, mean age 51, truck drivers	Within	Yes	1 h	No analysis carried out
Hollerit et al., 2021	iCO2	Sim (game)	78	No details	Correl-ational	No	7–8 min	No analysis carried out.
**User experience evaluations**								
Stillwater and Kurani, 2013	NA	Road	46	varying age and sex	Interview	N/A	4 weeks (2 weeks with gamification)	Positive evaluations of eco score goals; negative evaluations of leader board; spontaneously expressed driving with instant energy feedback was “like playing a game”
Rapp, 2016	The eco service	Road	16	Mixed gender; age 21–65	Interview	N/A	1 h	Positive rating of eco driving score and tracking scores over time; some rated leader board negatively.
Reiner and Reder, 2014	Social Driving App	Road	9	male, age 23–26	Questionnaire	N/A	30 min	Positive ratings of audio and visual feedback; System rated as usable and desirable overall
Bahadoor and Hosein, 2016	Project Drive	Road	6	age 20–30	Questionnaire	N/A	2 weeks (1 week with gamification)	Universal positive rating of badges/social feed (leader board) and fiscal rewards; majority positive rating for retrospective feedback.
Brouwer et al., 2015	NA	Sim	26	2f, mean age 51 years, truck drivers	Questionnaire	N/A	1 h	Positive ratings of eco-speed range, eco-driving performance score and live leader board
Vaezipour et al., 2016	NA	Attitude survey	34	mixed gender, age 19–61	Focus groups	N/A	N/A	Preference for personalized eco-driving feedback rather than a leader board; evidence of individual differences in preferences
Bingham and Shope, 2003	Streetwise	Sim (game)	19	if 15 m, aged 15 to 17 years	Questionnaire and focus groups.	N/A	50 min	Mixed attitude to game; perception of driving risk increased after playing
Rodrigues et al., 2015	NA	Sim	15	2f 13 m, aged 19–36 years	Questionnaire	N/A	6–10 min	Overall satisfaction high: usefulness, visual quality, ease of learning, ease of use, interactivity, fatigue, entertainment, effectiveness, presence, satisfaction.

Steinberger et al. (2017a) evaluated their adjunct system, “Coastmaster,” which encourages minimal use of the brake pedal during transitions from higher to lower speed limit areas. An ideal speed transition map is displayed visually and the driver is tasked with matching their speed to the transition target speed in real time. In a simulator, 32 male drivers aged 18–25 drove for 16 min with and without the system, with condition order counterbalanced. With the system activated inferential analyses showed reduced overall speed, reduced driving over the speed limit, and greater anticipation showed by more rapid speed lowering across speed limit transitions. There is much to commend the design and analysis of this study, with only the relatively small sample size counting against it. However, a further evaluation was presented in the same year. Steinberger et al. (2017b) showed that “Coastmaster” significantly lowered speed in a further simulator study with 24 male drivers. Taken together, these studies provide good evidence for the efficacy of the “Coastmaster” intervention.

Stillwater (2011) evaluated an adjunct system across 42 drivers on public roads over 1 year, finding mixed results. There was no overall benefit of the intervention for energy economy, but certain individuals who showed expertise in deciphering fuel economy feedback benefitted.

The remaining evaluation studies are flawed such that meaningful conclusions cannot be reached. Gunther et al. (2020) evaluated a smartphone-based adjunct system across 108 participants driving on public roads. However, the within-subjects study design was compromised by an invariant condition order. Vaezipour et al. (2019) assessed an adjunct system in 40 volunteers in a simulator, while McConky et al. (2018) also assessed an adjunct system in a simulator with 29 participants. However, in both studies the gamification condition included an extrinsic reward for improving the eco-score. Conflation of gamification and extrinsic rewards means these studies provide weak evidence that gamification can promote eco-driving. Ecker et al. (2011) evaluated a bespoke adjunct system in 36 drivers on roads in the city of Munich. While increased eco-driving score, reduced braking force, reduced time spent accelerating and increased coasting were claimed, the study design was unclear as a single baseline trip was mentioned in the text, yet the figures suggest challenges with multiple baseline measures. This lack of clarity detracts from the study findings.

Ando et al. (2010) evaluated a bespoke adjunct system across 50 participants driving on public roads, while Brouwer et al. (2015) evaluated an adjunct system in a truck driving simulator with 26 mostly male truck drivers. No statistical analysis of the data was carried out in either study. Two further studies are compromised by low statistical power due to a small sample size. Rapp (2016) compared across groups of size *n* = 8, while Soriguera and Miralles (2016) reported a pilot study with sample size *n* = 7. Overall, evaluations of eco-driving adjunct systems provide limited evidence for their effectiveness. However, this conclusion reflects absence of evidence rather than evidence of absence.

Hollerit et al. (2021) evaluated a game-based learning approach to eco-driving comprising a driving sim game designed to encourage eco-driving. However, while the system was evaluated across 2,455 users, only a small proportion (*n* = 78) played for more than 8 min, there was no statistical analysis, and the “improvement with time” study design did not include a control group. Overall, this study presents no evidence of the efficacy of this intervention for encouraging eco-driving, thus there remains an absence of evidence for whether a game-based learning approach can impact positively on eco-driving.

### User experience evaluations of the various gamified approaches

Six studies assessed user experiences of using adjunct systems (see Table 2). Four of these collected user impressions during road driving. Stillwater and Kurani (2013) recorded spontaneous reports that driving with instant energy feedback was “like playing a game,” with users challenging themselves to increase their fuel efficiency. However, social comparisons were rated less positively due to a confusing leader board display. Rapp (2016) recorded positive ratings of an eco-driving score and users were motivated by tracking their scores over time. Again, a leader board received mixed ratings due to a perception of unfairness as the top positioned drivers drove small-engined cars (although this could motivate switching to more economical cars – a desirable eco-driving outcome). A feature that displayed money saved via reduced fuel use was requested by users, and a desire was expressed for live feedback during driving, as found on other adjunct systems. Reiner and Reder (2014) recorded positive ratings of visual feedback including steering recommendations, an applause sound when eco-driving behavior was displayed, and a “puuuh” sound when not. Bahadoor and Hosein (2016) recorded users feeling “compelled” to see what badges their contacts had received, while a map displaying positive driving events (retrospective feedback) was positively rated. All users positively rated the possibility of receiving fiscal rewards for eco-driving. Brouwer et al. (2015) recorded positive evaluations of live feedback comprising an eco-speed range, a display of 1–5 stars and a leader board in a simulator study.

Vaezipour et al. (2016) had users evaluate design ideas for a smartphone-based adjunct system. Users preferred personalized eco-driving feedback rather than being shown other people’s performance, with a perception that leader boards should be optional, suggesting that systems should be customizable by the user. Users also highlighted the importance of matching in-app challenges to user skill, avoiding boredom for challenges that are too easy, or frustration for those that are too difficult. Overall users rated eco-driving scores and live feedback positively, while leader boards received mixed evaluations due to design issues.

Two game-based learning systems have undergone user experience evaluations (see Table 2). Bingham and Shope (2003) evaluated a web-based driving sim video game to a mixed reception. While all users enjoyed playing, still 70% did not wish to re-play, and under half would recommend the game to others. Many users wished for more feedback and would have liked a leader board. Rodrigues et al. (2015) evaluated a smartphone-based driving sim video game. There were positive ratings for ease of learning, entertainment, effectiveness and satisfaction. However, the small screen size and small buttons to control driving were rated negatively. Overall, user experience evaluations of game-based eco-driving interventions are mixed.

## Discussion

This systematized review evaluated 39 studies assessing gamification applied to eco-driving. Smartphone-based adjuncts to driving were the most frequently used format, followed by bespoke adjunct systems, and videogames designed to encourage eco-driving.

The first aim was to summarize the gamification elements researched in the context of encouraging eco-driving. The most popular of these were an eco-driving score which users were motivated to improve via competition against their own current score, or against others in a leader board format. Some systems included rewards for high scores such as badges (non-fiscal) or restaurant vouchers (fiscal) as well as encouraging longer term engagement via challenges, missions, quests or levels. Some imaginative gamified elements include happy/sad emoji displays, a car avatar character and a graphic showing a tree that becomes greener and lusher as eco-driving increases. Previous research has linked flow to higher road driving speed, theorizing that drivers may speed as a way of coping with the boredom of mundane driving (Stephens and Smith, 2022). This implies that any in-car game, from “I spy” to doing an audio quiz, could potentially ease boredom and reduce the temptation to speed, benefitting eco-driving. In keeping with this, some systems linked non-driving games to eco-driving such as snakes and ladders. Mention should be made of the “Meeco” system designed by Vara et al. (2011) which games drivers to use alternative transport or delay car journeys to less congested times of day.

The second aim was to assess the efficacy of gamification approaches for encouraging eco-driving. While numerous evaluation studies have been carried out (13 examples), the overall quality of this research has been poor. Recurring methodological problems include conflation of gamification with extrinsic rewards, invariant condition ordering, absence of inferential statistical data analysis and small sample size rendering apparent effects unreliable. With only a handful of well-conducted evaluation studies, it remains largely unsubstantiated whether gamification can be successfully applied to encourage eco-driving, and across what timescales. There is some evidence from research on public roads and driving simulators that an eco-score can be effective with Magaña and Muñoz-Organero (2015) finding fuel savings in road driving of 0.59 l/100 km, or 118 liters per year assuming annual driving of 20,000 km. Other researchers showed the “Coastmaster” system designed around a graphic interface guiding smoother transitions to lower speed limits demonstrably reduced overall speed and driving above speed limits in two well designed driving simulator studies (Steinberger et al., 2017a,b). A wide range of other gamification applications have been researched but there is insufficient evidence to adjudge their effectiveness.

A variety of theoretical approaches to gamification have been employed (see Table 1), however, choice of theoretical basis appears to have had little impact on intervention designs or likelihood of a successful outcome.

The third aim was to assess user experience evaluations of gamified approaches to eco-driving. Across eight studies users were willing to accept gamified systems for encouraging eco-driving, with no systems provoking strong negative user experience evaluations. Live feedback was rated positively with some users spontaneously reporting that systems providing instant feedback of fuel/energy use were “like games.” This suggests that for some drivers, the challenge of eco-driving can be fun, interesting and possibly flow inducing. Leader boards were not universally popular due to design issues. It is desirable for gamified systems to be easily customizable so that users can turn off parts that they do not like, rather than abandon them altogether. Adjunct systems used during driving were rated more positively than game-based systems, possibly because users are expected to engage with the latter in their spare time.

One limitation of this review is that no second researcher double checked that inclusion and exclusion criteria were applied fairly. On the other hand, consistent with open science practices, a spreadsheet detailing all included and excluded studies is available in the Supplementary material enabling scrutiny of decisions that were made.

Overall, this review concludes that gamification shows promise as a tool for encouraging eco-driving, but the literature is still developing and further adequately designed evaluation studies are required. Adjunct systems providing an eco-driving score are likely to be successful, although it is not possible to comment on the timescales over which gamification interventions are likely to be helpful. Consequently, there is no reason to limit further investigation to only those gamification elements supported by evidence to date. Rather, researchers should continue to evaluate a wide range of gamification approaches across a range of timescales.

## Author contributions

RS contributed to the conception and design of the study, compiled the Boolean search term, performed the database searches, reviewed materials, carried out supplementary statistical analysis, wrote the first draft of the manuscript, carried out manuscript revision, read, and approved the submitted version.

## Supplementary material

Supplementary Table 1 Excel Spreadsheet containing citations for all sources located in the searches, detailing the fate of each source (whether included or excluded), and where excluded, the stage at which exclusion occurred.
